# Maturity Stage Discrimination of *Camellia oleifera* Fruit Using Visible and Near-Infrared Hyperspectral Imaging

**DOI:** 10.3390/molecules27196318

**Published:** 2022-09-25

**Authors:** Hongzhe Jiang, Yilei Hu, Xuesong Jiang, Hongping Zhou

**Affiliations:** 1 Jiangsu Co-Innovation Center of Efficient Processing and Utilization of Forest Resources, Nanjing Forestry University, Nanjing 210037, China; 2 College of Mechanical and Electronic Engineering, Nanjing Forestry University, Nanjing 210037, China

**Keywords:** *Camellia oleifera* fruit, maturity, hyperspectral imaging, near-infrared, chemometrics

## Abstract

The maturity of *Camellia oleifera* fruit is one of the most important indicators to optimize the harvest day, which, in turn, results in a high yield and good quality of the produced *Camellia* oil. A hyperspectral imaging (HSI) system in the range of visible and near-infrared (400–1000 nm) was employed to assess the maturity stages of *Camellia oleifera* fruit. Hyperspectral images of 1000 samples, which were collected at five different maturity stages, were acquired. The spectrum of each sample was extracted from the identified region of interest (ROI) in each hyperspectral image. Spectral principal component analysis (PCA) revealed that the first three PCs showed potential for discriminating samples at different maturity stages. Two classification models, including partial least-squares discriminant analysis (PLS-DA) and principal component analysis discriminant analysis (PCA-DA), based on the raw or pre-processed full spectra, were developed, and performances were compared. Using a PLS-DA model, based on second-order (2nd) derivative pre-processed spectra, achieved the highest results of correct classification rates (CCRs) of 99.2%, 98.4%, and 97.6% in the calibration, cross-validation, and prediction sets, respectively. Key wavelengths selected by PC loadings, two-dimensional correlation spectroscopy (2D-COS), and the uninformative variable elimination and successive projections algorithm (UVE+SPA) were applied as inputs of the PLS-DA model, while UVE-SPA-PLS-DA built the optimal model with the highest CCR of 81.2% in terms of the prediction set. In a confusion matrix of the optimal simplified model, satisfactory sensitivity, specificity, and precision were acquired. Misclassification was likely to occur between samples at maturity stages two, three, and four. Overall, an HSI with effective selected variables, coupled with PLS-DA, could provide an accurate method and a reference simple system by which to rapidly discriminate the maturity stages of *Camellia oleifera* fruit samples.

## 1. Introduction

**Figure 1:**
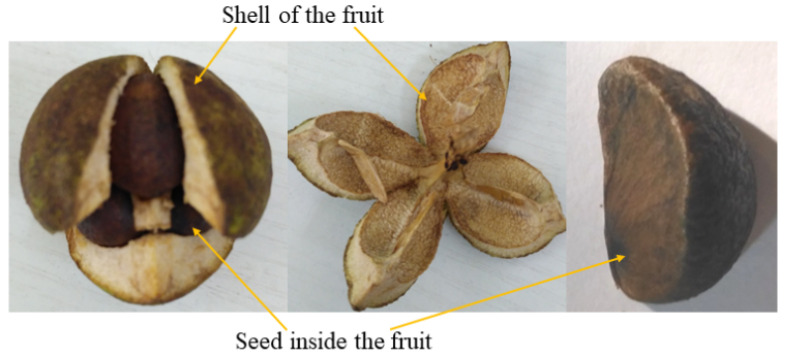
The internal structure of *Camellia oleifera* fruit.

*Camellia oleifera* Abel. (Theaceae), a traditional woody oil crop, is widely planted in southern China [1]. The edible oil extracted from the seeds inside *Camellia oleifera* fruit (shown in Figure 1) contains rich unsaturated fatty acids, tea polyphenols, vitamins, and camelliaside [2]. As consumers pay more attention to nutrition, health benefits are becoming the main factor in their choice of the *Camellia* oil. The maturity of the *Camellia oleifera* fruit is critical to the harvest; immature *Camellia oleifera* fruits will significantly lower the *Camellia* oil yields and reduce the overall oil quality [3]. The accurate identification of maturity stages is an effective measure used to guide the production, harvesting, and orchard management, and improve the oil yield and quality.

In fruit maturity estimation, several reliable indices, including total soluble solids (TSS), firmness, pH, titratable acidity (TA), dry matter (DM), color, or chlorophyll are generally employed [4]. Various methods have been applied to determine the maturity stages of agricultural products by researchers [5]; however, they are destructive in nature, time-consuming, and inapplicable to sorting. Traditionally, the maturity of *Camellia oleifera* fruits is identified by growers, according to the solar period. Another commonly accepted method is to remove the pericarp and observe the characteristics of the inner seeds. However, these methods are too subjective, time-consuming, or destructive.

Over the past decades, machine vision has been found to be applied for automatic and non-destructive assessment of the maturity levels of fruit, including bananas [6], melon [7], cape gooseberry [8], and oil palm [9]. Through a non-destructive imaging technique, fruit information on surface quality, including size, color, volume, and shape, can be extracted to evaluate the fruit’s maturity [10]. However, a single physical color or texture characteristic cannot incorporate all the factors responsible for maturity, and this would result in discrimination errors [11,12]. Additionally, the color or texture differences of samples at the intermediate adjacent maturity stages are not that significant. Near-infrared spectroscopy (NIRS) has been widely used as a reliable and rapid non-destructive technique that can be applied to quantifying molecules containing hydrogen atoms, such as water, alcohol, and other compounds formed by the C-H, N-H, O-H, and S-H groups [13]. For example, a portable NIRS instrument has been used to determine the maturity-related indexes of grapes, including pH, anthocyanin concentration, and sugar [14]. NIRS has great advantages including a short measuring time with limited sample preparation, allowing several constituents to be evaluated at the same time. At present, NIRS can be used to detect the internal qualities of various fruits; however, single-point detection can hardly represent the whole sample. To date, there have been few studies related to identifying the maturity stages of *Camellia oleifera* fruit samples using imaging or NIRS.

Hyperspectral imaging (HSI) has been a powerful tool for the comprehensive analysis of agricultural and food production that integrates spectroscopy and machine vision to collect both spectral and spatial information from one target [15,16,17,18]. HSI represents the spectra at each pixel of an image, utilizing the advantages of near-infrared spectroscopy, conventional imaging, and even multispectral imaging techniques. Recently, HSI has been applied to classifying the maturity stages of various agriculture products, such as persimmon [19], strawberry [20], peanut [21], and tomato [22], which showed the great potential use for fruit maturity indices or stage assessments. To our knowledge, no endeavors have as yet been carried out to evaluate the maturity of woody oil fruit.

The overall objective of this study was to explore the potential of the HSI technique for assessing the maturity stages of *Camellia oleifera* fruit. The specific objectives were: (1) to extract the full spectra, representing samples from identified characteristic regions of interest (ROIs); (2) to conduct principal component analysis (PCA) on extracted spectra and hyperspectral images to establish preliminary distinguishability; (3) to develop classification models in view of various pre-processing stages, partial least square-discriminant analysis (PLS-DA), and discriminant analysis, coupled with principal component analysis (PCA-DA) modeling methods; (4) to select key variables using PC loadings, two-dimensional correlation spectroscopy (2D-COS), and uninformative variable elimination and successive projections algorithm (UVE+SPA); (5) to build simplified models using selected key wavelengths and compare the performances; (6) to evaluate the performance and determine an optimal simplified model.

## 2. Material and Methods

### 2.1. Sample Preparation

**Figure 2:**
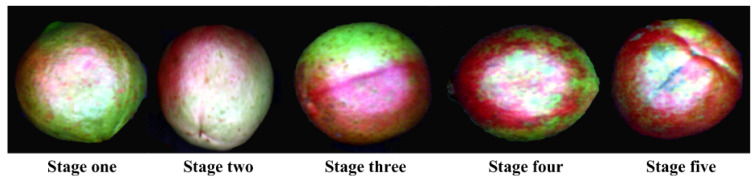
Pseudo-color images showing the five maturity stages of typical *Camellia oleifera* fruit samples.

*Camellia oleifera* fruit samples were hand-harvested every week during the 2020 harvest season (September to October) from the Jinhang specialized *Camellia* cooperative in Jiangning District in Nanjing City, Jiangsu Province, China (31.68° N, 118.89° E). *Camellia* trees were randomly selected for collecting samples. Two hundred fruit samples at each maturity stage (S1: stage 1, samples picked on 25 September 2020; S2: stage 2, samples picked on 30 September 2020; S3: samples picked in stage 3, on 5 October 2020; S4: stage 4, samples picked on 13 October 2020; S5: stage 5, samples picked on 20 October 2020) without any visual defects or bruises were collected from a single row in the orchard on each harvesting day (Figure 2). After that, the samples were stored in an incubator covered with ice bags and transported to the Jiangsu agricultural and forestry products spectral imaging detection laboratory at Nanjing Forestry University, Nanjing City, Jiangsu Province, China. The *Camellia oleifera* fruit samples were restored to room temperature, set at about 25 °C, then hyperspectral image acquisition and physicochemical experiments were conducted. Therefore, a total of 1000 samples across five different maturity stages were collected. Among each set, samples were randomly chosen to be used for further physicochemical measurements. In total, all the 1000 samples were grouped randomly, based on a ratio of 3:1. The 750 samples were randomly divided into a calibration set, while the other 250 samples were employed for an independent prediction set. The pseudo-color images of typical *Camellia oleifera* fruit samples are presented in Figure 2; the peel color did not show a clear change from stage one to stage five. Several cracked and overripe *Camellia oleifera* fruit samples in the control group were also collected on 27 October 2020.

### 2.2. Hyperspectral Image Acquisition and Calibration

After the samples were equilibrated to room temperature for 20 min, the hyperspectral images were then acquired to eliminate the potential temperature effects. Since the fruit is an irregular ellipsoid, the samples were placed in individual stable directions onto the conveyor, prior to image acquisition. A hyperspectral image of each *Camellia oleifera* fruit was collected using a VNIR (336.2–1092.5 nm) spectrograph (GaiaField-V10E-AZ4, Sichuan Dualix Spectral Image Technology Co., Ltd., Chengdu, China), with a 16-bit charged-couple device (CCD) camera, a light source consisting of twelve 50-W lamps, a dome coated with Teflon for uniform light, a conveyor belt (HSIA-CSD800) propelled by a servo motor, and a computer (Lenovo Yangtian A4600t, Lenovo Group Ltd., Beijing, China). The distance between the surface of the sample being imaged and the hyperspectral camera was 300 mm. The optimal data acquisition parameters were determined after multiple pre-experiments. The exposure time of the camera was set as 1.2 ms and the speed of the conveyor belt was 0.6014 nm/s. The diagram and other detailed parameters (e.g. the spectral resolution was 2.8 nm and image resolution was 800 × 664 pixels) of this HSI system can be found in a previous work [23].

To eliminate the effects of uneven illumination and dark current, black-and-white calibration was conducted on raw image, *I*_r_, using Equation (1), as follows:

(1) $$I_{c}=(I_{r}-\;I_{d})/(I_{w}\;-\;I_{d})\;\times \;100\%$$

where *I*_r_ indicates the raw hyperspectral image, *I*_c_ is the calibrated hyperspectral image, *I*_w_ expresses the image collected from a reference Teflon plate (~100% reflectance), and *I*_d_ denoted the dark reference image acquired by covering the lens with its own opaque cap (~0% reflectance).

### 2.3. Reference Analysis

On each sampling day, ten *Camellia oleifera* fruit samples were randomly selected to perform the reference analysis. The height and diameters of the samples were measured using a Vernier caliper (111-101v-10G, Guilin Guanglu Measuring Instrument Co., Ltd.). The pericarp of the *Camellia* fruits was prepared by cutting them into small pieces and drying them using a drying-oven (DHG-9101-2SA, Changzhou Langyue Instrument Manufacturing Co., Ltd.) at 105 °C to establish a constant weight, to calculate the moisture level. Mass measurements for fruits and seeds were conducted using an electronic balance (BSM-220.4, Shanghai Zhuojing Electronic Technology Co., Ltd.) and then recorded. The seed yield was calculated by dividing the seed mass by the fruit mass. The *Camellia* oil content (oil extracted from the internal seeds of the fruit) was determined using a soxhlet fat analyzer (NAI-ZFCDY-6Z, Shanghai Naai Precision Instrument Co., Ltd.) using the Soxhlet extraction method, as in a previously published analysis [24].

### 2.4. Extraction of Spectra

**Figure 3:**
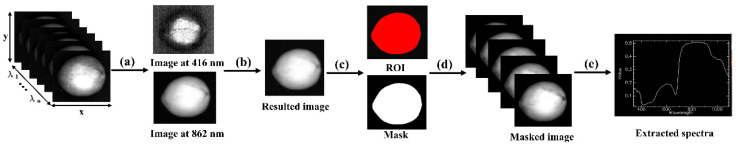
The main steps involved in the segmentation of hyperspectral images. (**a**) Selecting 416 nm and 862 nm images; (**b**) band math; (**c**) binarization; (**d**) applying the mask; (**e**) extracting the spectra of a *Camellia oleifera* fruit sample.

The region of interest (ROI) was first predefined to extract spectral information from the calibrated hyperspectral images. To select the whole *Camellia oleifera* fruit sample as the ROI, image segmentation was performed to remove the background by creating a mask. The main steps involved in image segmentation are displayed in Figure 3. Since the reflectance intensity of the image at 862 nm was the highest quality and the image at 416 nm was the lowest quality (Figure 3a), these were chosen. Band math (Figure 3b) was carried out by subtracting the image at 416 nm from the image at 862 nm to create a greyscale image with high contrast between the sample and background. In the resulting image, a binary mask image was generated by setting a reflectance threshold of 0.1 (Figure 3c). By applying the mask image, the sample and the background were separated, and the pixel value in the background was 0 (Figure 3d). The mean extracted spectrum representing each sample was finally identified by averaging the spectral responses of all the pixels in the masked hyperspectral image (Figure 3e).

### 2.5. Spectral Pre-Processing

Spectra that are extracted from hyperspectral images contain noise, potential interference of particle size, and the scattering of light. Chemometric pre-processing is broadly performed to reduce the undesired information from the spectra prior to modeling, and to eliminate the influence of interference signals [25]. In the present work, four pre-processing methods were available and were adopted based on their practicality and popularity of use in spectral analysis. Standard normal variate (SNV), normalization, and first-order (1st) and second-order (2nd) derivatives were individually applied to the spectra. SNV was used for correcting scatter and removing the slope fluctuation from the spectra [26]. Derivatives were applied to separate the existing overlapping absorption bands and eliminate baseline offsets [27]. In this study, Savitzky-Golay-based derivatives were implemented with a fixed window of 7 and a second-order polynomial. Normalization was conducted by transforming the spectral vector into the unit length to eliminate multiplicative spectral effects [28]. In this study, max-min normalization was applied to each spectrum. The above pre-processing stages were carried out by the Unscrambler X10.1 software (CAMO, Trondheim, Norway).

Principal component analysis (PCA) was generated in an effort to reduce the data dimensionality and seek feature variables. Briefly, PCA transforms the original variables (*m*) into a new set of *m* variables, named principal components (PCs). These PCs are all linear combinations of the original variables [29]. The original variables take specific values in PC space, called PC scores. Visual analysis of the samples’ distribution with PCs can be conducted in the score plot, and the loading lines are beneficial for seeing the weighted coefficients for each variable. In this study, dimensionality reduction and feature wavelength selection were carried out by PCA. All the pre-processing methods were applied, using in-house codes in MATLAB 2013b software (MathWorks, Natick, MA, USA).

### 2.6. Modeling Methods and Assessment

The partial least-squares discriminant analysis (PLS-DA) algorithm is a classical and efficient chemometrics method for qualitative analysis, based on the principles and characteristics of partial least-squares regression (PLSR). PLS-DA predicts the class for each input, and the smallest number of transformed new variables, called latent variables (LVs), with the minimum prediction error of the sum of squares (PRESS), will be chosen [30]. In this study, ten-fold cross-validation with Venetian blinds, combined with the PLS1 algorithm, was employed to determine the number of LVs in the calibration set.

The discriminant analysis, coupled with the principal component analysis (PCA-DA) algorithm, is a PCA-based analysis method that was also employed in this study. In PCA-DA, the spectra were first analyzed by PCA to investigate the presence of categories inside the sample population. After that, discriminant analysis (DA) was conducted, based on un-rotated PC scores. The number of PCs was optimized in the feature subspace, and DA were subsequently used in the linear classification in the subspace. PCA-DA considered the class membership information provided by the auxiliary matrix, in the form of codes, when constructing the factors. Therefore, it had an effective discriminative ability and improved the validity and effectiveness of the model [31].

As mentioned above, samples were randomly separated into two sets, based on a ratio of 3:1, from which 750 samples were chosen as a calibration set; the remaining 250 samples were applied as a prediction set. To evaluate the performance of the established models in terms of classification, the correct classification rate (CCR) in the calibration set, cross-validation set, and prediction set were calculated using the following equation:

CCR = *N*_1_/*N*_2_ *×* 100% (2)

where *N*_1_ indicates the number of correctly classified samples at each maturity, and *N*_2_ denotes the number of all the samples.

Sensitivity, specificity, and precision are commonly applied terms to further assess the performance of established models to describe the accuracy of a classification test. Sensitivity, specificity, and predictions were calculated using the following equations:

(3) $$\mathrm{Sensitivity}=\frac{\mathrm{TP}}{\mathrm{TP}+\mathrm{FN}}$$

(4) $$\mathrm{Specificity}=\frac{\mathrm{TN}}{\mathrm{TN}+\mathrm{FP}}$$

(5) $$\mathrm{Precision}=\frac{\mathrm{TP}}{\mathrm{TP}+\mathrm{FP}}$$

where TP represents the true positive, TN means the true negative, FN denotes the false negative, and FP indicates the false positive. All the data modeling and models’ assessments were carried out using Toolboxes for the MATLAB 2013b software (MathWorks, Natick, MA, USA).

### 2.7. Dimensionality Reduction Methods

A large amount of redundant information exists in this context, due to the continuous hyperspectral bands, and most of them are useless and should be excluded when dealing with assessment model establishment. Uninformative variable elimination (UVE) is a method for removing useless information and reducing the data dimension, based on a stability analysis of the regression coefficient in PLSR. Briefly, noise matrices are added artificially into the original variables, and a closed form of the PLSR model was obtained, containing the original and artificial variables [32]. According to the criteria, based on regression coefficients, no original variables that were more important than the artificial variables would be eliminated [33].

The successive projections algorithm (SPA) is a forward variable selection algorithm for multivariable calibration. SPA applies a simple projection operation in vector space to obtain subsets of spectral variables with small collinearity [34]. Generally, SPA works in an iterative way by the successive orthogonal projection on the remaining variables. The variables with the maximum projection values on the orthogonal subspace of the previously selected variables will finally be employed. In this study, UVE was first used to select the informative wavelengths, then SPA followed to select those variables with minimum redundant information from the informative ones (UVE+SPA) [35].

The 2D-COS method is a very popular mathematical method being employed when seeking spectral differences among different sets of spectra under one certain external perturbation. The 2D-COS explores the very subtle spectral changes that are hardly detected by conventional 1D spectral analysis. In generalized 2D-COS, the perturbation can be concentration, pressure, temperature, etc. [36]. After that, the dynamic original data will be recast into two orthogonal representations in the 2D-COS analysis, i.e., the synchronous and asynchronous corrections. The synchronous 2D-COS spectrum is employed to characterize the similarity of the spectral variations at each variable. The strong variation will lead to an autopeak at a specific variable, which will be selected. In this study, maturity was applied as the external perturbation, and the characteristic maturity-related wavelengths were observed in synchronous 2D-COS. All the wavelength selection methods were conducted using Toolboxes in the MATLAB software.

## 3. Results and Discussion

### 3.1. Statistical Characterization of Samples at Different Maturity Stages

The mean values of the physicochemical attributes measured for the five different maturity stages, as well as the control of the overripe stages, are summarized in Table 1. Numerical changes can be seen in the various physicochemical properties in different stages. More specifically, the height and diameter increased regularly during the progressing maturity from S1 to S5 of the *Camellia oleifera* fruit. Fruit mass and *Camellia* oil content also increased during the ripening process of the *Camellia oleifera* fruit, which was consistent with our cognition. The *Camellia* oil content reached its peak of 35.54% at S5, which indicated that an early picking will significantly affect the total oil output. Seed mass, seed yield, and pericarp moisture tended to grow with the increase in maturity stages; however, the parameters in S5 were lower than those in S4. The reason might be that complex internal component changes occurred, including starch, water, fat, etc.

### 3.2. Spectral Profiles

**Figure 4:**
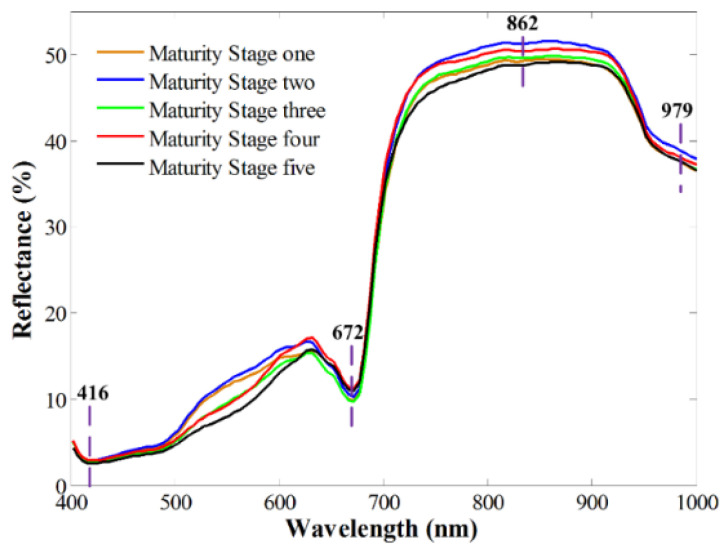
Average spectra of *Camellia oleifera* fruits at each maturity stage.

Mean spectral profiles, extracted from the hyperspectral images of samples at different maturity stages, are shown in Figure 4. A visual inspection of the spectra extracted from acquiring the hyperspectral images showed that spectra in two end regions of wavelengths were rather noisy, with a low signal-to-noise ratio (SNR) in the two spectral regions. Therefore, the two spectral regions of 336.2 to 397.5 nm and 1005.7 to 1092.5 nm were excluded. The remaining 119 bands in the spectral range of 400–1000 nm were further applied for developing classification models in this study. All the samples presented similar spectral patterns; low reflectance intensity was shown in a spectral range of 400–700 nm, while high reflectance values were 700–1000 nm. A slight intensity difference can be seen in the mean spectra of *Camellia oleifera* fruit samples at different maturity stages. This difference is related to the slight changes caused by an interaction of light and samples, as maturity increased. As presented in Figure 2, the color traits of samples at different maturity stages were slightly different. The spectra presented a low reflectance near the spectral band at 416 nm, which could be attributed to Soret absorption [37]. Another reflectance valley appeared at 672 nm, which was related to the chlorophyll content in forest fruit [38]. The band at 979 nm could be ascribed to the second overtone of the O-H stretching mode of water or carbohydrates [39]. A minor valley at 862 nm was associated with the first overtone of O-H stretching. Further usage of chemometrics will be beneficial to tease out the spectra in different groups.

### 3.3. Principal Component Analysis

**Figure 5:**
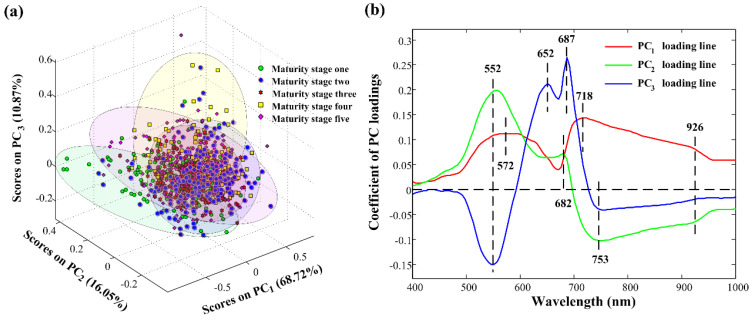
PCA results. (**a**) Score plot; (**b**) PC loading lines. PCA, principal component analysis; PC, principal component.

PCA was applied for differentiating *Camellia oleifera* fruit samples at different maturity stages. Raw full spectra at 400–1000 nm were taken. PC_1_, PC_2_, and PC_3_ explained 68.72%, 16.05%, and 10.87% of the total variance, respectively. These first three PCs, accounting for a total of above 95% of the total variation, were considered for this study because the majority of spectral information can be explained by them. The score plot of the combination of PC_1_, PC_2_, and PC_3_ in Figure 5a showed a general separated trend. Five clusters were generated, with some overlap among them. The main reason was that PCA did not perform well when a large number of multi-class samples were included [40]. This PC score space showed the potential for good classification results. What is more, detailed PC loading lines presented how the spectral information at all wavelengths contributed to classifying samples into different maturity stages. In the PC loading lines in Figure 5b, eight characteristic wavelengths (552 nm, 572 nm, 652 nm, 682 nm, 687 nm, 718 nm, 753 nm, and 926 nm) resulting from pronounced peaks and valleys of the first three PC loading lines were identified. These maturity-related wavelengths were retained and were used further in classifying the samples into different maturity stages.

**Figure 6:**
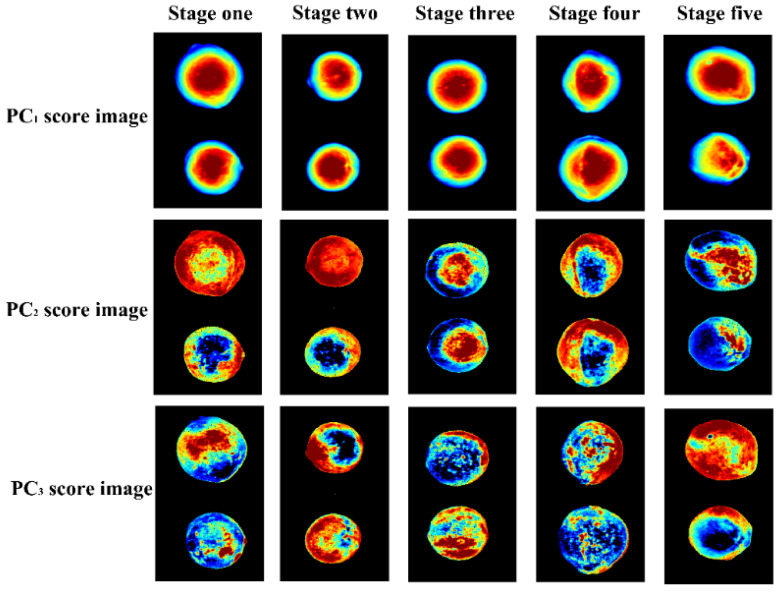
The first three PC-score images of the samples at five maturity stages. PC, principal component.

As PC_1_, PC_2_, and PC_3_ were able to represent a total of 95.64% of variances, Figure 6 provides the corresponding first three PC score images of the samples at five different maturity stages. In the figure, pixels have different PC score values and are shown in different colors. Not very notable differences in PC_1_ score images among the different groups in terms of color could be observed. Slight differences were observed in PC_2_ and PC_3_ score images. A high misclassification rate was generated due to their similar spectral curves. The PC score image results were consistent with the spectral PCA, showing that having a large number of multi-class samples included in the set would lead to a great deal of overlap. The PC score images indicated that the cluster and visualization of samples in different groups were slightly different. In order to further explore the feasibility of spectral information, it was still necessary to develop calibration models for discriminating the maturity stages of the samples.

### 3.4. Model Development, Based on Full Spectra

Different pre-processing activities were applied to the raw full spectra, and both raw and pre-processed spectra were employed to establish the PLS-DA and PCA-DA models, to determine the optimal pre-processing and modeling methods. All the results are shown in Table 2. Cross-validation was carried out in the calibration set using the Venetian blinds method. As was shown, the spectral classification accuracy was greater than 79.5% regardless of the modeling and preprocessing methods, which indicated that the established models can easily distinguish the samples of different maturity stages. Selections of the optimal numbers of LVs in the PLS-DA model and PCs in the PCA-DA model were based on the lowest error in the cross-validation step. The PLS-DA classification models achieved overall higher CCRs in the prediction sets (82.8% vs. 80.8%, 95.6% vs. 83.2%, 95.6% vs. 91.2%, 88.0% vs. 79.6%, and 97.6% vs. 91.6%) than PCA-DA models, which indicated that PLS-DA was more suitable than PCA-DA in distinguishing the samples. As for the pre-processing techniques, the method of the 2nd derivative performed best, regardless of the modeling methods. The classification accuracy was 99.2% for the calibration set, 98.4% for the cross-validation set, and 97.6% for the prediction set in the PLS-DA model, developed by the 2nd derivative pre-processed spectra. Therefore, 2nd derivative preprocessing, combined with the PLS-DA modeling method, was adopted in further analysis.

### 3.5. Effective Wavelengths Selection

Hyperspectral images contain an immense amount of data, which significantly limited their practical application and data analysis. High-dimensional full spectra suffered wavelength redundancy and co-linearity, which caused low practicability and complex models. From the perspective of model application, it is of great importance to develop robust models on a small number of variables. Effective wavelength selection will contribute to reducing the instrument costs and computation time [41]. In the present study, PC loadings (in Section 3.3), 2DCOS, UVE and SPA were utilized to select the most effective wavelengths with the most information for discriminating the different maturity stages of *Camellia oleifera* fruit samples.

**Figure 7:**
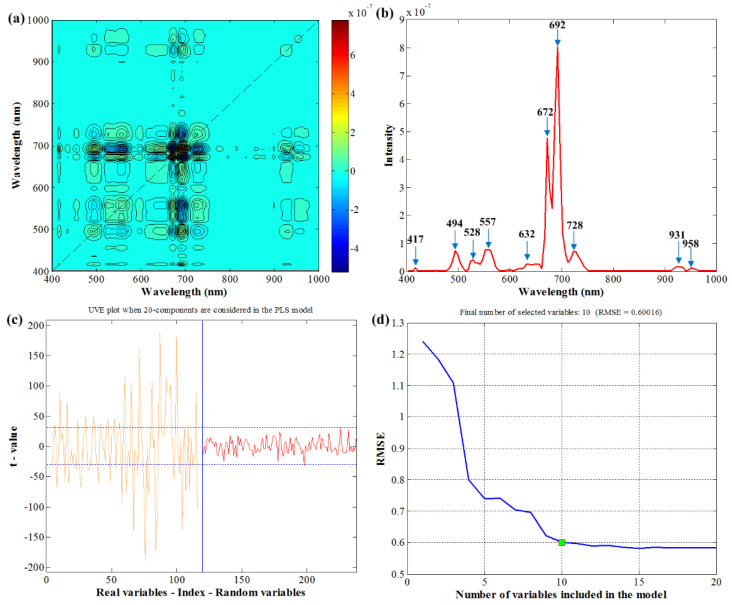
Wavelengths selection steps. (**a**) The synchronous 2D correlation spectra; (**b**) spectrum in a diagonal line in synchronous 2D correlation spectra; (**c**) the stability of wavelengths and random variables in UVE; (**d**) the changing trend of RMSE with the increase in selected wavelengths by SPA. 2D, two-dimensional; RMSE, root mean squares error; SPA, successive projections algorithm.

By using 2D-COS, the synchronous 2D correlation spectra of samples at five different maturity stages are shown in Figure 7a. The synchronous spectrum reveals the presence of autopeaks and crosspeaks; ten wavelengths, including 417 nm, 494 nm, 528 nm, 557 nm, 632 nm, 672 nm, 692 nm, 728 nm, 931 nm, and 958 nm can be observed on the diagonal line extracted in Figure 7b. In the UVE process, 119 random variables (the same number with original variables) were added to the dataset. Figure 7c shows the stability curve of the wavelength variables and random variables. The curve on the left in yellow refers to the stability of the original variables, while the right curve represents the random variables. Two dotted lines indicate the cutoff thresholds, and variables where the stability lies within the dotted lines were eliminated. As a result, 62 wavelengths were retained using UVE, then SPA was conducted following UVE. As the number of selected wavelengths increased in Figure 7d, the root mean squares error (RMSE) showed a gradual downward trend. When 10 variables were achieved, the RMSE value reached an optimal value so that only 10 wavelengths were retained by UVE+SPA. All the selected wavelengths by various methods are listed in Table 3, and most of the variables selected by UVE+SPA were in the range of 700–1000 nm, which was different from the ones selected by 2D-COS and PC loadings.

### 3.6. Establishment of PLS-DA Models, Based on Selected Wavelengths

In this work, PLS-DA was adopted to develop a calibration model for the maturity-stage classification of *Camellia oleifera* fruit samples. Three PLS-DA models, based on the variables selected by PC loadings, 2DCOS, and UVE+SPA, were developed. The detailed classification results of optimized models built using only the optimal wavelengths are displayed in Table 4. Compared to the models developed using the full spectra, the performances were degraded. The CCR in the prediction set of the optimal models decreased from 97.6% to 81.2%. However, the combination of UVE and SPA achieved this best result and sped up the modeling procedure. The wavelengths selected by PC loadings and 2DCOS performed poorly, with a CCR of only about 55.5% in the prediction set.

To further explore the UVE-SPA-PLS-DA model in identifying different maturity stages, the predictive ability of this optimized model is presented in Table 5. In the confusion matrix, the samples at maturity stage one were clearly discriminated, with a CCR of 98.0%, sensitivity of 0.98, specificity of 0.95, and precision of 0.83. It also can be seen from the confusion matrix that the classification in S5 also obtained a satisfactory result for the CCR of 88.0%. The model seemed to find it difficult when classifying samples in maturity stage four, with a CCR of only 66.0%. Additionally, samples in S3 or S2 were misclassified to their adjacent neighbor. The result could be explained by the close similarity of spectra between S2, S3, and S4. Overall, a multi-spectral system using the ten wavelengths previously selected by UVE+SPA will be strongly recommended, which will make more sense due to the availability, simplicity, and low cost.

## 4. Conclusions

The identification of the maturity stages of *Camellia oleifera* fruit is conducive to optimizing the harvest time. In this study, HSI was applied as an alternative way to discriminate the different maturity stages of *Camellia oleifera* fruit samples. As can be shown in this study, numerous physicochemical attributes, including fruit mass and *Camellia* oil content, increased as the maturity stages increased. The spectra of a total of 1000 samples at five maturity stages were obtained in a spectral range of 400–1000 nm. The spectral PCA indicated that the first three PCs had the potential to be effective in classifying samples at different stages. PLS-DA and PCA-DA, based on the spectra, were pre-processed with various methods that were applied to develop the classification models; the results showed that the PLS-DA model developed by the 2nd derivative spectra gave the best prediction CCR of 97.6%. The PC loading, 2DCOS, and UVE+SPA methods were individually employed to select key maturity-related wavelengths from the full wavelengths. The UVE-SPA-PLS-DA model showed satisfactory performance for classifying the different maturity stages, with an 81.2% CCR in the prediction set. Therefore, this simplified model was chosen and could be applied. The knowledge gained in this work could be incorporated into the forestry fruit industry to produce high-quality products. Further studies are still needed to facilitate its adoption on an industrial scale.

## Figures and Tables

**Table 1:** Statistical results of the mean values and standard deviation (SD) for the physicochemical properties (*n* = 10).

Maturity Stages	Height (mm)	Diameter (mm)	Fruit Mass (g)	Seeds Mass (g)	Seeds Yield (%)	Oil Content (%)	Pericarp Moisture (%)
S1	40.32 ± 0.35	40.08 ± 0.15	28.40 ± 1.32	10.27 ± 0.43	36.16 ± 3.13	22.31 ± 0.93	70.19 ± 4.56
S2	40.45 ± 0.32	40.21 ± 0.14	27.32 ± 1.56	10.36 ± 0.36	37.92 ± 4.77	24.03 ± 0.73	70.23 ± 5.69
S3	40.53 ± 0.33	40.39 ± 0.15	29.60 ± 2.03	11.30 ± 0.35	38.18 ± 4.31	27.46 ± 1.02	68.97 ± 5.35
S4	41.12 ± 0.36	40.96 ± 0.18	30.21 ± 2.13	12.98 ± 0.42	42.97 ± 5.10	32.27 ± 1.12	69.12 ± 6.21
S5	41.24 ± 0.35	41.03 ± 0.25	30.64 ± 1.89	11.85 ± 0.41	38.67 ± 3.95	35.54 ± 1.13	68.65 ± 5.36
Control	41.16 ± 0.42	41.65 ± 0.21	30.55 ± 2.77	12.64 ± 0.48	41.37 ± 4.32	35.06 ± 0.84	66.39 ± 3.13

**Table 2:** Results of PLS-DA and PCA-DA models based on the full spectra using various pre-processing techniques.

Modeling Methods	Pre-Processings	Correction Classification Rate			Parameters
		Calibration Set	Cross-Validation Set	Prediction Set	
PLS-DA	None	93.9%	92.9%	82.8%	LV = 18
	SNV	97.9%	96.5%	95.6%	LV = 19
	Normalization	98.7%	96.7%	95.6%	LV = 19
	1st derivative	95.2%	93.6%	88.0%	LV = 19
	2nd derivative	99.2%	98.4%	97.6%	LV = 16
PCA-DA	None	90.3%	88.5%	80.8%	PC = 20
	SNV	89.1%	87.1%	83.2%	PC = 20
	Normalization	95.7%	94.7%	91.2%	PC = 20
	1st derivative	86.4%	84.0%	79.6%	PC = 20
	2nd derivative	94.9%	93.9%	91.6%	PC = 18

**Table 3:** Wavelength selection by different methods.

Methods	Numbers	Selected Wavelengths (nm)
PC loadings	8	552, 572, 652, 682, 687, 718, 753, 926
2DCOS	10	417, 494, 528, 557, 632, 672, 692, 728, 931, 958
UVE+SPA	10	572, 622, 652, 753, 774, 821, 862, 873, 894, 963

**Table 4:** Performance of multi-spectral PLSR models using selected wavelengths.

Model.	LVs	Correction Classification Rate (%)		
		Calibration Set	Cross-Validation Set	Prediction Set
PC-PLS-DA	7	57.9	56.1	55.6
2DCOS-PLS-DA	9	68.8	66.9	54.0
UVE-SPA-PLS-DA	9	83.6	82.1	81.2

**Table 5:** Confusion matrix of prediction set for UVE-SPA-PLS-DA model.

Actual Stages	Predicted Stages					CCR	Sensitivity	Specificity	Precision
	S1	S2	S3	S4	S5				
S1	49	1	0	0	0	98.0%	0.98	0.95	0.83
S2	8	37	1	1	3	74.0%	0.74	0.95	0.80
S3	2	2	40	4	2	80.0%	0.80	0.94	0.78
S4	0	4	7	33	6	66.0%	0.66	0.97	0.85
S5	0	2	3	1	44	88.0%	0.88	0.94	0.80

## Data Availability

The data presented in this study are available on request from the authors.
